# The Moderating Effect of Age on Low-Income Students' Relationships With Mentors and Their Self-Efficacy Since COVID-19

**DOI:** 10.3389/fpsyt.2022.800385

**Published:** 2022-04-08

**Authors:** Jaewon Lee, Jennifer Allen, Hyejung Lim, Gyuhyun Choi, Jiyu Jung

**Affiliations:** ^1^School of Social Welfare, Inha University, Incheon, South Korea; ^2^School of Social Work, Michigan State University, East Lansing, MI, United States; ^3^School of Education, Korea University, Seoul, South Korea; ^4^Integrative Arts Therapy, Dongduk Women's University, Seoul, South Korea; ^5^Korea Development Bank Foundation, Seoul, South Korea

**Keywords:** low-income students, self-efficacy, mentors, age, COVID-19

## Abstract

**Purposes:**

This study investigates the moderating effect of age on the association between relationship with mentors and self-efficacy among low-income students.

**Methods:**

A total of 255 low-income middle and high school students participated. The PROCESS macro 3.4 for Statistical Product and Service Solutions was employed to test the moderating effect.

**Results:**

Quality of relationship between mentors and mentees was positively associated with mentees' self-efficacy. Students' age significantly moderated the association between quality of the relationship with mentors and self-efficacy.

**Discussion:**

It is important to expand mentorship programs for low-income students during the COVID-19 pandemic in order to foster high self-efficacy among adolescents. Recruitment of high quality mentors and additional factors that may be helpful to a good relationship between mentees and mentors, such as mentor training, mentor screening, and mentor-mentee matching, should be prioritized to improve self-efficacy among low-income students since COVID-19. Early opportunities for mentoring from high quality mentors is particularly important to increase self-efficacy among younger students such as middle school students.

## Introduction

High self-efficacy is an important predictor of various positive outcomes among adolescents, such as increased positive thinking and affect, decreased depression, and decreased internalizing and externalizing problems (1–3). Overall, self-efficacy beliefs rise from adolescence into adulthood, but self-efficacy beliefs may decrease before again rising in adolescence, making it an important time to bolster adolescents' self-efficacy (4). One intervention that may help to bolster self-efficacy among adolescents are mentorship programs (5–7), where adolescents are paired with a trained mentor who supports the adolescent socially or academically, for example. Quality of the mentor-mentee relationship may affect adolescents' mentorship-related outcomes (6); however, some studies only examine adolescents' participation in a mentorship program, without examining perceived quality or effectiveness. Further, an age difference may exist in the association between the quality of the mentor-mentee relationship and self-efficacy between middle school students and high school students. Thus, more research is needed on the relationship between adolescent mentees' perceived quality of their relationship with their mentor on the development of mentees' self-efficacy in the context of age.

### Positive Impacts of Mentorship for Low Income Students

Across several studies, mentorship programs have found to positively affect low- income students in diverse ways (8–13). In one study, low-income students in South Korea who were satisfied with their participation in a mentorship program showed lower levels of depression than those students who were less satisfied with the program (10). The remaining studies focused primarily on academic-related outcomes. Among eighth and ninth grade students attending a school in a low-socioeconomic status (SES) community, participants in a mentor outreach program were up to 25% more likely to have a positive perception of science compared to students who did not participate in the program (13). In another study of perceived at-risk high school students in a school district where 65% of students qualified for free or reduced-price lunch, students who were randomly assigned to a school staff mentor had a higher sense of school belonging, higher perceived teacher support, and were less likely to have been disciplined at school than students who did not receive mentorship (9).

Moreover, among a student body where 85% were eligible for free lunch at school, students who reported having a natural mentor in their lives reported fewer school absences, higher expectations of school success for themselves, and a higher sense of school belonging, and these effects were intensified if the student reported a higher number of mentors in their lives (12). Additionally, researchers paired teenagers living at or below 125% of U.S. federal poverty guidelines with mentors, but they found no significant differences in self-esteem, grades, school attendance, or school disciplinary infractions; the authors posited that the lack of significant effects may be related to the relatively short median length of the mentor-mentee relationship, as well as the lack of a measure examining the quality of the mentor-mentee relationship (11). Last, at-risk middle school students paired with a school faculty or staff mentor were sent to the office significantly less often and showed improvements in attitude toward school (8). These findings suggest that, overall, participation in a mentorship program can be associated with numerous positive outcomes for low-income middle- and high-school students.

### Age Differences

In a review of the literature on mentoring, the researchers noted that mentorship programs designed for adolescents rarely distinguish between the needs of older and younger adolescents (14). Such age-related differences in needs may impact the effectiveness and positive outcomes in mentorship for adolescents of different ages. For example, because social support is especially important during the transition period of early adolescence, simply having support from a mentor may be associated with particularly positive outcomes (14). Additionally, in one study of community- and school-based mentorship program, the age of the mentee was associated with the mentor's report of the quality of the mentor-mentee relationship, with mentors with middle or high school aged mentees reporting a less close or supportive relationship than those with elementary-aged mentees (15). Last, a systematic review of school-based mentoring among adolescents identified two studies that looked at age differences in their outcomes of interest (16). In the first study which analyzed a mentorship program for students aged 9–15 years, the older students had improved school attendance, but the younger students did not (17). In the second study, which examined students in grades 4 through 8, students aged <12 years had lower truancy after participating in the mentorship program than did students above age 12 (18). These contradictory findings, as well as the lack of studies looking at age differences in the outcomes of mentorship programs, demonstrate that this is a research area that warrants more attention.

### Self-Efficacy During Adolescence

Findings regarding the development of self-efficacy in adolescence vary, perhaps due to differences in defining and categorizing self-efficacy (4). As teenagers develop, according to Schunk et al. (4), they “form more stable and integrated views of their capabilities, values, and attributes” (4). Some studies found that by 7th grade, adolescents' perceptions of their own competence decline before increasing as they age (4). However, while some studies have found a decline in mathematics self-efficacy in adolescence, other studies have found increases in both language and mathematics self-efficacy (4). In another study comparing the self-image and self-efficacy of adolescent girls and boys, the researchers found that girls tended to have higher academic and regulatory self-efficacy than boys, while boys had higher emotional self-efficacy (19). More research is needed in adolescent self-efficacy, particularly using more standardized measures.

### Impact of Self-Efficacy on COVID-19-Related Outcomes

Research has recently emerged on the impact of self-efficacy as a buffer against negative outcomes related to the COVID-19 pandemic among adolescents and adults, underlining the importance of high self-efficacy (20). In a group of adolescents during a COVID-19-related lockdown period in Italy, positive self-efficacy predicted subjective wellbeing and positive coping, and subjective wellbeing partially mediated the relationship between self-efficacy and positive coping during the lockdown (20). In a longitudinal study of children aged 11–16, an increase in mental health symptoms during the pandemic was buffering by high self-efficacy (21). Additionally, among adults during an 8-week period of COVID-19-related confinement in France, self-efficacy remained fairly stable during this period, and greater self-efficacy was positively related to positive affect (and vice versa for negative affect), as well as positively related to work performance during this period (22). Self-efficacy also positively predicted mental health during the COVID-19 pandemic among adults in Turkey (23).

### Mentorship and Self-Efficacy

A few studies have examined the relationship between mentoring and mentees' self-efficacy among adolescents, college students and graduate students (5–7). In a sample of middle school students who participated in a 10-week after-school mentorship program, quantitative analyses did not show large changes in students' self-efficacy, but in qualitative interviews, the students expressed that they did feel greater self-efficacy than before the program (7). Additionally, among American Indian college students who participated in a paid mentorship program for 8 sessions, participants did report higher self-efficacy after participation than before (5). Last, among Hispanic graduate students who reported having a mentor during their program, mentored students had significantly higher academic self-efficacy, and 3 mentorship related variables accounted for 24% of the variance in academic self-efficacy: Having a mentor, having a faculty mentor, and effectiveness of the mentor (6). Thus, evidence suggests that participation in a mentorship program has a positive effect on mentees' self-efficacy, but more research is needed among adolescents of different ages and regarding the effect of the quality of the mentor-mentee relationship on self-efficacy.

### The Current Study

Mentorship programs have been regarded as an important way to bring about high self-efficacy and positive behavioral changes among students (8–13). However, little attention has been given to students' relationships with mentors, which is a key factor to determine the quality of a mentorship program. Low-income students in particular might have experienced reduced attention from their parents or caregivers since COVID-19 because it has become more difficult to earn a living during the crisis due to economic recession (24). That is, mentors have played an important role in empowering students in low-income families since COVID-19. This study focuses on the quality of the relationship between mentees and mentors in a mentorship program rather than the quality of the mentorship program itself. Given that a good relationship with mentors can be helpful to develop self-efficacy in adolescence (7), the current study explores an association between the quality of students' relationship with mentors and self-efficacy among low-income students since COVID-19. In addition, age may influence the association as younger students (i.e., middle school students) are more open to others' advice and feedback compared to older students (i.e., high school students) who have their own thoughts and may be developing independence and an increased reliance on peers (25). Thus, this study also investigates the moderating effect of age on the association between relationship with mentors and self-efficacy among low-income students.

## Methods

### Participants and Sampling

Participants in this study included a targeted, nationwide sample of middle and high school students who had engaged in a mentorship program provided by a Non-profit organization, the Korea Development Bank [KDB] Foundation. The mentorship program was only available to students from low-income families. We reached out to the 264 middle and high school students enrolled in the mentorship program to participate in our study in April 2021. Participants responded to an online survey via Google Forms. The questionnaire was first developed by the research team, and then it was reviewed by a teacher in public school and social workers. Based upon their feedback, the questionnaires were refined to minimize potential risks of the participation and to help participants clearly understand the survey questions. To access the survey, a text message including a link for the survey was distributed to potential participants. Further, both students and their caregivers received a consent form before the participation so that they were able to select whether they consented to respond to the survey. Depending on their responses, nine low-income students were not included in the final sample because either student or their caregiver(s) declined their participation. Thus, a total of 255 low-income students participated and they received a $5 gift card as a reward for participating. As the current study did not collect any private information such as name, address, and the like, this study was approved by the Institutional Review Board (#210216-2A).

### Measures

#### Self-Efficacy

Self-efficacy in this study was measured by the General Self-Efficacy Scale (GSE) developed by Schwarzer et al. (26). This scale consists of ten items as follows: “I can always manage to solve difficult problems if I try hard enough”; “If someone opposes me, I can find the means and ways to get what I want”; “It is easy for me to stick to my aims and accomplish my goals”; “I am confident that I could deal efficiently with unexpected events”; “Thanks to my resourcefulness, I know how to handle unforeseen situations”; “I can solve most problems if I invest the necessary effort”; “I can remain calm when facing difficulties because I can rely on my coping abilities”; “When I am confronted with a problem, I can usually find several solutions”; “If I am in trouble, I can usually think of a solution”; and “I can usually handle whatever comes my way”. The ten items have a four-point Likert-type scale, ranging from 1 to 4 (1 = Not at all true; 2 = Hardly true; 3 = Moderately true; 4 = Exactly true). The score of each item was summed and a higher score demonstrated higher levels of self-efficacy. The reliability and validity of this scale were checked in 23 countries, including South Korea (26). The Cronbach's α of the self-efficacy scale used in the current study was 0.90.

#### Relationships With Mentors

Low-income students responded to a series of questions regarding the quality of the relationship between themselves and their mentors. The relationship with mentors refers to the closeness of the relationship between mentor and mentee and how well they are able to maintain the relationship without challenges. Five items were used to measure the quality of the relationships between mentees and mentors: “I think that my mentor values me as much as I value him or her”; “My mentor respects me”; “I found it easy to get emotionally close to my mentor”; “I found it easy to trust my mentor completely”; and “I am happy with my mentor and the relationship”. Each item was rated on a five-point Likert-type scale, and response options were ranged from 1 = strongly disagree to 5 = strongly agree. A total score was calculated based on the sum of all items, and a higher score demonstrated a higher quality relationship between mentors and mentees. This scale had a Cronbach's alpha of 0.75.

#### Age

Asian countries use a different way to calculate age, which is different from one's international age. In these countries, a newborn is considered 1 year old at birth. Thus, to calculate participants' international age, 1 year should be deducted from the age given.

#### Control Variables

Gender and academic performance (e.g., letter grades of A, B, C, etc.) were controlled for in this study. Further, parents' educational attainment was included. If both their mothers and fathers had higher education, those were categorized in the parental higher education group, while others were placed in the Non-higher education group.

### Analysis Strategies

**Figure 1:**
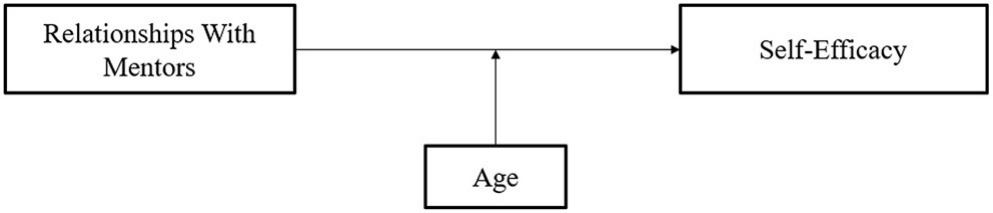
Research framework.

The PROCESS macro 3.4 for Statistical Product and Service Solutions (SPSS) was employed to test the moderating effect of age on the association between the quality of the relationship between mentors and mentees and low-income students' self-efficacy. A bootstrap approach developed by Preacher and Hayes was applied to analyze the data, and five-thousand iterations of the bootstrapping method were conducted at the 95% confidence intervals. A research framework is shown in Figure 1.

## Results

Descriptive statistics are reported in Table 1. Scores of self-efficacy and quality of the relationship between mentors and mentees were 29.8 and 20.2, respectively. Respondents' average age was 17.4 years old, or an international age of 16.4 years. Almost half of the sample were girls (49.4%). Given that their average academic performance was 7.7, respondents' average letter grade was about C. 39.6% of participants had both mothers and fathers who had completed higher education.

**Table 1:** Descriptive statistics.

**Variables**	**% or Mean (SD)**
Self-efficacy	29.83 (5.58)
Relationships with mentors	20.16 (3.44)
Age	17.36 (1.75)
Gender (girl)	49.4%
Academic performance	7.66 (3.73)
Parents' educational attainment	39.6%

**Figure 2:**
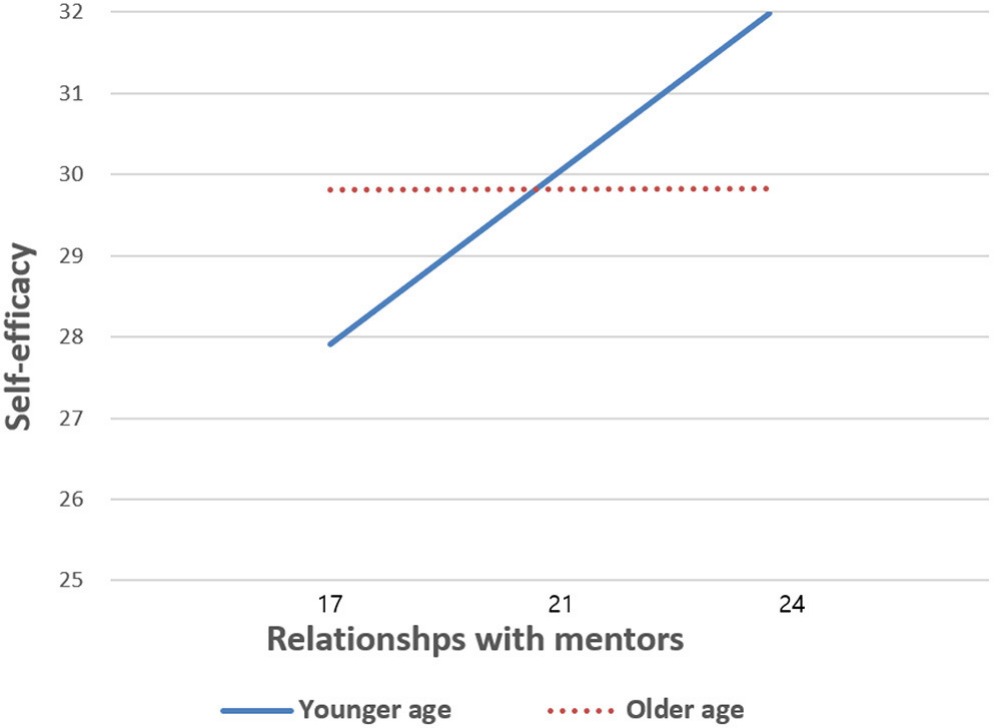
Moderating effect of age on the association between relationships with mentors and self-efficacy among low-income students.

A moderating effect of age on the relationship between quality of relationship between mentor and mentee and participants' self-efficacy was confirmed in Table 2. Students' age significantly moderated the association between quality of the relationship with mentors and self-efficacy (β = −0.12, *p* < 0.01). Further, age itself was statistically related to self-efficacy (β = 2.35, *p* < 0.01). Quality of relationship between mentors and mentees was positively associated with mentees' self-efficacy (β = 2.32, *p* < 0.01). Being a boy and having greater academic performance were positively related to self-efficacy (β = −1.34, *p* < 0.05; β = 0.28, *p* < 0.01, respectively). Figure 2 shows the moderating effect of age on the relationship among low-income students. In this study, high school students showed constant levels of self-efficacy, regardless of whether they had lower or higher scores for the quality of the relationship with their mentors (29.81 vs. 29.82). However, younger, middle school students reported a greater gap in self-efficacy between those with a lower quality of relationship with their mentor (27.91) and those with a higher quality of relationship with their mentor (31.98). In other words, high school students' self-efficacy was not influenced by the quality of the relationship with their mentor, while self-efficacy was greatly influenced by the quality of the relationship with their mentor among middle school students.

**Table 2:** Moderating effects of age on self-efficacy using SPSS process.

**Variables**		
(Constant)	−19.11 (18.73)	
Relationships with mentors	2.32 (0.92)	^*^
Gender(girl)	−1.34 (0.67)	^*^
Academic performance	0.28 (0.09)	^**^
Parents' educational attainment	1.19 (0.68)	
**Moderator**		
Age	2.35 (1.08)	^*^
**Moderating effect**		
Relationships with mentors^*^Age	−0.12 (0.05)	^*^

* *p < 0.05*.

** *p < 0.01*.

## Discussion

This study focuses on low-income students' self-efficacy since COVID-19. The current study's findings explored the association between the quality of the relationship between mentors and mentees and mentees' self-efficacy, and also tested the moderating effect of age on the association among middle and high school students who have grown up in low-income families. Low-income students who participated in a mentorship program and maintained a good relationship with mentors were more likely to develop higher levels of self-efficacy. In addition, there was a significant moderating effect of age on the association between the quality of the relationship between mentors and mentees and mentees' self-efficacy since COVID-19. That is, self-efficacy among younger low-income students was more greatly influenced by the quality of the relationship with their mentors.

Findings in the current study indicated that the quality of the relationship between mentors and mentees was related to self-efficacy among low-income students. This is consistent with previous studies showing that positive social relationships or social support are beneficial to increase self-efficacy among adolescents (27, 28). However, there is no evidence about the relationship among low-income students. Further, most studies have primarily considered the impact of the mentorship program in general, rather than focusing on the relationships between mentees and mentors (11, 12). Thus, this study shed a light on understanding the importance of the quality of the relationship between students and their mentors. Given that low-income students are less likely to have a chance to develop and cultivate their self-efficacy (29), perhaps due to limited resources, lower support from their caregivers and economic challenges, the relationships between mentees who come from low-income families and mentors are critical to improve self-efficacy among low-income students. Particularly, since COVID-19, low-income students might have decreased opportunities for learning and health, such as fewer after school learning programs being provided in-person, as well as fewer school meals being provided, because of the risks of spreading COVID-19 (30, 31). As a result, these students might be more isolated and disconnected from education and self-development, leading to a fear of failure and mental health problems (32). That is, they have very constrained environments to develop their self-efficacy since COVID-19. Self-efficacy might be one of the most important factors to address stresses from the pandemic situation, particularly for students who are greatly exposed to academic pressure and have had reduced opportunities to experience a variety of outdoor activities due to school closures (33). Mentorship programs have proven to help individuals to develop self-efficacy and to psychologically empower students (6–8). However, previous studies have mainly just addressed the relationship between participation in a mentorship program itself and self-efficacy or mental health (6, 7, 9, 12). Quality of mentorship is partially determined by the relationship between mentee and mentor, as the effects of mentorship are partially derived from warm feedback and advice from mentors. Despite the importance of quality of the mentor-mentee relationship, few studies focus on the relationships with mentors and self-efficacy. In particular, little is known about this association among low-income students since COVID-19.

This study found that the quality of the mentee-mentor relationship among low-income adolescents was important for self-efficacy since COVID-19. Provided that students have reduced time to communicate with teachers and friends in school in-person during parts of the COVID-19 pandemic (34), an opportunity to build a relationship with a mentor and opportunities to talk with them is beneficial to improve self-efficacy, as mentors may emotionally support students' decisions and future careers. Therefore, it is important to expand mentorship programs for low-income students during the COVID-19 pandemic in order to foster high self-efficacy among adolescents. However, rather than simply providing more mentorship programs, mentors who can create a close relationship with their mentee based on rapport-building should be recruited, as the relationship between mentees and mentors is crucial to self-efficacy. Thus, recruitment of high quality mentors and additional factors that may be helpful to a good relationship between mentees and mentors, such as mentor training, mentor screening, and mentor-mentee matching (15, 35), should be prioritized to improve self-efficacy among low-income students since COVID-19.

The current study also revealed that age moderated the association between the quality of the mentor-mentee relationship and mentees' self-efficacy. Middle school students were more likely to improve their self-efficacy if a greater quality relationship with their mentors had been maintained. On the other hand, self-efficacy of high school students in low-income families was not influenced by the quality of the relationship with their mentors. As individuals age, they may be more likely to realize their current situation and economic status and the impacts they may have on their lives (36, 37). High school students in low-income households may be more aware of the economic difficulties that may limit their daily life compared to middle school students. As a result, empowerment or support from mentors might not older children's or adolescents' self-efficacy because they already understand the potential constraints of their socioeconomic status. Self-efficacy is closely related to confidence in one's behaviors and belief in one's abilities to accomplish tasks (38). However, high school students who already aware of their families' financial challenges might have less hope to enhance their conditions, leading to no changes in self-efficacy through mentoring. However, low-income middle school students who might be relatively less mature than high schoolers might be more strongly influenced by mentors' feedback and support. In particular, students have encountered diverse troubles among their friends and families since COVID-19, such as increased family conflict and decreased friend support (39, 40), but they may not have an adequate outlet to relieve their stresses or address issues in their lives because of social distancing. In addition, economic difficulties might negatively impact a sense of confidence among low-income middle school students and such a challenge may interrupt the development of self-efficacy. A good relationship with mentors can buffer against anti-social behaviors and serve as a guide to face challenges. For middle school students, such assistance is beneficial to improve their capability to address problems by themselves, resulting in higher levels of self-efficacy. Given the effect of age on the association between the quality of the mentor-mentee relationship and self-efficacy in this study, early opportunities for mentoring from high quality mentors is particularly important to increase self-efficacy among younger students such as middle school students. Further, low-income middle schoolers should be prioritized to receive mentorship.

This study provides new evidence about the importance of age on the association between the quality of the mentor-mentee relationship and self-efficacy among low-income students. During the coronavirus pandemic, fewer resources are available for students due to social distancing measures and economic recession (41), but when it comes to prioritizing support, low-income middle school students should be prioritized to receive mentoring based on a high-quality mentor-mentee relationship to improve their self-efficacy, which is one of the most important factors influencing achievement in one's life (42). Particularly, low-income middle school students who can increase their self-efficacy through a high-quality relationship with mentors may be more likely to succeed in their lives, perhaps leading to breaking free from poverty or low-income status in adulthood.

This study newly considers the role of age on the association between the quality of the mentor-mentee relationship and self-efficacy among low-income middle and high school students. However, interpretations should be considered in the context of limitations. First, this study was conducted in South Korea, which might have different cultural and economic environments compared to other nations. Thus, the types and numbers of mentorship programs available and characteristics of low-income students might be different from those in different countries. Second, this study only compared middle school students with high school students, so that the moderating effect of age on the association between the quality of the mentor-mentee relationship and mentees' self-efficacy is not considered elementary school students. We recommend that future studies expand the ages of participants including elementary schoolers. Third, there might be a variety of factors that influence self-efficacy. As this study included limited control variables due to limitations of the online survey method, we suggest that more variables that may affect the self-efficacy should be included in future studies. Fourth, students' academic performance is closely related to self-efficacy and another interesting story may turn up if we focus on academic performance. Thus, we recommend that future studies consider academic performance as a mediator or independent variable.

## Data Availability Statement

The raw data supporting the conclusions of this article will be made available by the authors, without undue reservation.

## Ethics Statement

The Institutional Review Board of Inha University approved this study (#210216-2A). Written informed consent to participate in this study was provided by the participants' legal guardian/next of kin.

## Author Contributions

All authors have read and agreed to the published version of the manuscript, contributed to the article, and approved the submitted version.

## Conflict of Interest

The authors declare that the research was conducted in the absence of any commercial or financial relationships that could be construed as a potential conflict of interest.

## Publisher's Note

All claims expressed in this article are solely those of the authors and do not necessarily represent those of their affiliated organizations, or those of the publisher, the editors and the reviewers. Any product that may be evaluated in this article, or claim that may be made by its manufacturer, is not guaranteed or endorsed by the publisher.
